# Rwandan primary healthcare providers’ perception of their capability in the diagnostic practice

**DOI:** 10.4102/phcfm.v12i1.2197

**Published:** 2020-09-16

**Authors:** Ditte L. Weber, Vincent K. Cubaka, Per Kallestrup, Susanne Reventlow, Michael Schriver

**Affiliations:** 1 Department of Planning, Faculty of IT and Design, Aalborg University, Aalborg, Denmark; 2 Department of Primary Health Care, School of Medicine and Pharmacy, University of Rwanda, Kigali, Rwanda; 3 Center for Global Health, Department of Public Health, Faculty of Health, Aarhus University, Aarhus, Denmark; 4 Department of Public Health, Faculty of Health and Medical Sciences, Copenhagen University, Copenhagen, Denmark; 5 Department of Public Health, Faculty of Health, Aarhus University, Aarhus, Denmark

**Keywords:** diagnostic capability, healthcare providers, primary health care, health centre, Rwanda

## Abstract

**Background:**

Skill-mix imbalance is a global concern for primary healthcare in low-income countries. In Rwanda, primary healthcare facilities (health centres, HCs) are predominantly led by nurses. They have to diagnose a multitude of health complaints. Whether they feel capable of undertaking this responsibility has yet to be explored.

**Aim:**

This study explored how healthcare providers (HPs) at Rwandan HCs perceived their capability in the diagnostic practice.

**Setting:**

Rural and urban HCs in Muhanga district, Rwanda.

**Method:**

Qualitative, semi-structured interviews with nurses and clinical officers, and observations of consultations were made. Findings were analysed thematically.

**Results:**

Rwandan HPs were confident in their competences to perform diagnostic procedures although nurses felt that the responsibilities lay beyond their professional training. Clinical officers believed that their professional training prepared them to function competently and autonomously in the diagnostic practice, although all HPs experienced a high dependency on medical history taking, physical examination and laboratory tests for reaching a diagnosis. Resource constraints (time, rooms and laboratory tests) were seen as a barrier to perform diagnostic tasks optimally, and HPs experienced in-service training and supervision as insufficient. They increased their diagnostic competences through work experience, self-learning and supportive peer collaboration.

**Conclusion:**

Clinical officers perceived themselves as capable in the diagnostic practice. Nurses may compensate for insufficient school training through in-service learning opportunities and feel capable in the diagnostic practice. Formative mentorship schemes and tailored education may prove valuable, but further research on how to improve HPs’ diagnostic capability in Rwanda’s primary healthcare sector is needed.

## Introduction

A health system’s ability to provide quality healthcare services depends on an adequate ‘skill-mix’ of healthcare providers (HPs). This implies that there is a sufficient number of HPs, who are competent to carry out tasks that match the healthcare needs of the population.^1,2^

Skill-mix imbalance is a major global challenge, particularly in low-income countries (LICs), where there is a shortage of trained and competent HPs and limited resources, such as medical equipment and time.^3^

A common approach to tackle skill-mix imbalances is to optimise the use of the existing workforce by expanding their tasks and roles, hereinafter referred to as ‘task delegation’.^1,2^ Task delegation is commonly done in primary healthcare (PHC) in LICs where nurses and other HPs deliver services that fall outside the scope of their routine training, and carry out tasks that would have ideally been performed by physicians.^1,2^ Yet, task delegation is not always combined with adequate training consistent with these responsibilities.^1^ This is a major concern in many countries in sub-Saharan Africa (SSA), especially in rural health facilities.^2,4^

Diagnostic accuracy is a key element of effective treatment and provision of healthcare services.^5^ Evidence suggests a low diagnostic accuracy among primary HPs in out-patient departments (OPD) at health centres (HCs), dispensaries and first-level hospitals in several SSA countries.^6,7,8,9,10^ Here, limited or unavailable para-clinical diagnostic tools, such as laboratory tests, may lead to diagnostic uncertainties. In this context, diagnostic accuracy is usually determined by a thorough and systematic medical history (MH) and physical examination (PE),^11,12,13^ and requires skills in interpreting and synthesising symptoms and signs.^14^ In addition, algorithms (clinical guidelines) are used to guide the diagnostic decision-making in resource-constrained clinical settings.^12,14^

### Primary healthcare in Rwanda

In Rwanda, 85% of healthcare needs are addressed at PHC facilities,^15^ where clinical tasks and decisions are managed by non-physician clinicians.^16,17^ HC handle more than 90% of all out-patient visits in the health system.^18^

HPs working in the OPD of HCs manage a wide range of diseases and carry out comprehensive and complex tasks. They conduct consultations and diagnostic elucidation, decide which laboratory tests to do, who to treat and how, when to follow-up, who to refer to higher level facilities and who to send home.^15,17^ These tasks differ from nurses’ tasks in hospitals, which primarily consists of providing bed-side care and assisting physicians.^18^

HC are primarily staffed by nurses with an A2-level (A2-nurses) who make up 90% of all nurses in Rwanda. Since 1962, A2-nurses were trained through a 3-year secondary school education, with a focus on nursing care.^15,16,19^ As the quality of service delivery by A2-nurses was considered insufficient, the training was disrupted in 2007.^20,21^ Nurses with an A1-level (A1-nurses) were trained since 1996 and A2-nurses are being upgraded to A1-level to create a health workforce that mainly consists of A1-level or higher.^15,19^ The A1-level is 3 years of nursing school after secondary school, leading to an advanced, certified diploma in nursing.^15,16,19^ A1-nurses still represent < 10% of the total pool of nurses.^15,16^ Nurses with an A0-level are primarily trained to work in hospitals, HPs’ education or administration and few work at HCs.^15,16,19^

To address the shortage of skilled primary HPs, an initiative was taken to educate clinical officers (COs). Clinical officers are extensively and exclusively trained in community health and clinical medicine at the PHC level and have been suggested to achieve higher diagnostic accuracy than nurses. Therefore, many countries in SSA are educating and scaling up this HP cadre.^8,14,22^

The CO programme in Rwanda started in 2011 and was intended to constitute a 4-year programme post-secondary school, or an 18-month bridging programme to upgrade A1-nurses. The aim was to produce enough COs to employ in all HCs in Rwanda, but among the 176 who graduated, anecdotal evidence suggests only few work at HCs. Recently, the bridging programme was discontinued, and the 4-year programme never began because of resource limitations.^22^

The aim of this study was to explore how nurses (A2 and A1) and COs working in the OPD at Rwandan HCs perceived their capability to carry out procedures important for reaching a diagnosis.

## Research methods and design

### Study design

An exploratory study design was used to investigate the perceived capability in the diagnostic practice among HC providers.^23^ An iterative, pragmatic field approach was used, where HPs’ perceptions were explored within their everyday practice and context (the OPD at HCs).^23^

### Setting

There are approximately 495 HCs in Rwanda, containing nearly 60% of all beds in the health system. Health centres handle services such as HIV-care, TB-care, maternal- and antenatal care, child immunisation, hospitalisation In-Patient Department (IPD), laboratory work and OPD. Nurses often shift between these services.^18^ This study focusses on nurses and COs working in the OPD, as their tasks are complex and manifold, and require comprehensive skills in diagnosing patients.

Merely seven COs were identified as working at HCs in this study. Among those, one CO worked at a HC in the district of Muhanga, where the main data collection tool place. Few nurses working at HCs have an A1-level. This means that the HCs and the OPD service are primarily staffed by A2-nurses.

### Study population and sampling strategy

The sampling of three interviews with each HP cadre, A1-nurses, A2-nurses and COs (nine interviews in total), was guided by the principle of information power. The sample size was not determined prior to data collection but was assessed as sufficient after a comprehensive data analysis of the insights provided by participants.^24^ Arguments for a larger sample were that the study aim was guided by an exploratory approach and that we analysed cases across three different HP cadres. The three interviews with each HP cadre were deemed sufficient because of the quality of dialogue between researchers and participants. This was considered strong and clear, as interviews were guided by a focussed, semi-structured interview guide developed by health professional researchers, and interviews were carried out by a journalist and communication specialist. This provided rich insights into the scope of the study. In addition, characteristics of participants were highly specific to the study aim, as they concern the specific target groups (A2-nurses, A1-nurses and COs) with their variations of the experienced phenomenon (perceived diagnostic capability).

Observations of HPs were based on convenience sampling,^24^ as merely two A1-nurses and nine A2-nurses were available for observation.

HPs were excluded if they (1) had not done their HP education in Rwanda, as we explore the educational background (A2-nurses, A1-nurses and COs) for Rwandan HPs; (2) had an additional education in clinical health science, as this may affect HP’s diagnostic capabilities; (3) were newly educated (less than the probation period of 6 months), as we explore the importance of clinical experience. Both sexes were represented, and age ranged from 33 to 58 years (cf. Table 1).

**TABLE 1:** Participant characteristics by nine interviewed healthcare providers at Rwandan health centres.

Participant	1	2	3	4	5	6	7	8	9
HP cadre	A2	A2	A2	A1	A1	A1	CO	CO	CO
HC type	Rural	Semi-urban	Urban	Semi-urban	Semi-rural	Urban	Rural	Urban	Rural
Sex	Male	Male	Male	Male	Female	Female	Female	Female	Male
Age	33	34	35	58	51	49	38	38	37
Years, OPD	4–9	4–9	10+	4–9	4–9	10+	10+	4–9	0–3
Days, OPD per week	2–4	5+	2–4	2–4	2–4	2–4	5+	2–4	2–4
Patients per day	20–34	50+	50+	20–34	50+	20–34	50+	50+	50+
Referral (no diagnosis) per week	< 1	< 1	< 1	Daily	2–4	2–4	< 1	2–4	< 1
Send home (no diagnosis) per week	< 1	< 1	< 1	< 1	< 1	< 1	< 1	< 1	N/A
Hours, OPD daily	4–7	8+	8+	4–7	8+	4–7	4–7	4–7	4–7

OPD, out-patient departments; CO, clinical officer; HPs, healthcare providers, HC, health centre; N/A, Not available; A2, A2-level in general nursing; A1, A1-level in general nursing.

HPs from multiple sites were included to get a broad empirical data set and ensure that findings were not site-specific. A mix of rural, semi-rural, urban and semi-urban HCs were selected, with an assumption of rural–urban variations in terms of HP cadres, facility- and human resources, referral patterns and diagnostic accuracy.

HC were mainly chosen from the district of Muhanga, which is considered a rural district. Two HCs from two districts outside Muhanga (Nyamirambo and Kamonyi) were included to obtain perceptions from COs, as there was only one CO working at HCs in Muhanga.

### Data collection

As the aim was to explore HPs’ perceptions of their capability in the diagnostic practice, individual in-depth interviews with a semi-structured interview guide were used as the main empirical data collection method. The interviews were carried out at HCs in Rwanda’s national language, ‘Kinyarwanda’, to make participants comfortable and ensure that they were able to express themselves most adequately. The journalist and communication specialist carried out the interviews, as the principal investigator (PI) did not speak Kinyarwanda. The PI and the interviewer collaborated closely about the conduct and interpretation of the interviews. Interviews focussed on the diagnostic process; how work function and educational background match; how competent HPs felt to perform diagnostically; and educational and in-service opportunities for professional development.

Observations of HPs working in the OPD-service were carried out by the PI to substantiate the interview findings. To ensure similarity of observations, an observation sheet was used. This focussed on MH-taking, PE, use of algorithms, non-verbal patient–provider communication and how long consultation lasted. In addition, participants filled out an information sheet with HP characteristics.

To pretest the interview guide and observation sheet, two interviews with A2-nurses and two interviews with A1-nurses, as well as observations of the same nurses, were carried out in June 2015.

After adjusting the interview guide and observation sheet, data were collected from September to December 2015. Interviews were recorded and each interview lasted 60–100 min. The interview guide was adapted for every interview according to answers, inherent to the iterative approach.^25^ As some questions regarding educational training were specific to A1-nurses and COs, the interview guide differed between HP cadres.

Data from interviews were transcribed verbatim and translated from Kinyarwanda to English by a professional local translator and checked by a second person who went through samples. Notes made during observations were turned into descriptive accounts immediately after returning from the site. Complete records of all research material were kept encrypted in a password-protected computer.

### Data analysis

A systematic, iterative and thematic data analysis was conducted.^20,25^ Firstly, the PI read the raw data material to familiarise with data and identify initial themes. Then, a comprehensive coding process using the qualitative data analysis software MAXQDA was performed. The coding process was inductive to let participants’ perspectives lead the analysis and happened simultaneously with data collection as part of an iterative approach. Coding of interview data was done separately by the PI and the interviewer. Observational data were coded by the PI. Following this, codes were harmonised and refined, and structured into themes in a thematic framework. This process was done by four of the researchers. Following this, the PI used the identified thematic framework to re-code the raw data material. This was done to ensure that preliminary themes were consistent with empirical data. Emerging themes were structured further into categories, and quotes and descriptions were provided to explain findings. Lastly, a conceptual framework (Empowerment in the workplace) was used to discuss the findings, as the concept is equivalent to perceived capability in the diagnostic practice. The concept has been widely used in HP research.^21,26,27^

### Ethical consideration

Ethical and research clearance was granted by the Institutional Review Board of the College of Medicine and Health Sciences at the University of Rwanda (CHMS/IRB/216/2015). Informed consent was obtained from participants, who were kept anonymous during data analysis and interpretation.

## Results

### Perceived diagnostic capability by healthcare providers

The exploration of HP’s perceptions of their capability in the diagnostic practice revealed seven emerging and related themes: dependency on diagnostic tools; the match between work function and education; educational training opportunities; perceived competences to perform diagnostic tasks; resource constraints; in-service opportunities for professional development; and supportive relations at work. These are presented in the following paragraphs.

Table 1 displays the key characteristics and behaviours of HPs related to the diagnostic practice. This information was used to support HPs’ perspectives throughout the results section. Table 1 illustrates that all HPs had at least 4–9 years of work experience in the OPD and spent minimum 4–7 hours in the OPD per day, 2–4 times a week. All HPs claimed to send patients home without a diagnosis less than once per week. Some referred to the district hospital less than once per week, others 2–4 times a week and one A1-nurse daily. Most HPs saw more than 50 patients daily.

### Level of dependency on diagnostic tools in the diagnostic practice

All HPs found it crucial to do the procedure of MH-taking and PE adequately and in a detailed manner to reach a diagnosis, and observations revealed that most nurses did a full systematic PE. An A1-nurse explained:

‘Once you have done the anamnesis and physical exam appropriately, the patient can’t go back home with the problem they had, but once you did not do them well, a patient may go back home with the problem they have because you did not identify it.’ (HP5, A1-nurse, female, 51 yr.)

A CO expressed that they make autonomous decisions, whereas nurse decisions are supervised:

‘When we were studying [*nursing*] they taught us that we have to work under a medical doctor’s supervision, do only something you are recommended to. Contrary, in clinical [*CO training*] we learn to make clinical decisions.’ (HP9, CO, male, 37 yr.)

Confirming this, an A1-nurse mentioned that authorities help them as they cannot work and make decisions on their own, and an A2-nurse explained that nurses should follow algorithms that guide clinical decisions while physicians can work independently of them.

However, nurses did not always find algorithms valuable, describing how sometimes, when following algorithms, they were not able to identify a disease. An A1-nurse expressed that using algorithms creates a risk of replacing HPs’ own knowledge, making care standardised rather than individualised. While one A1-nurse stated that it may be valuable to use algorithms when their training in a particular domain is insufficient, several HPs from all cadres expressed that they relied more on their own judgement and competences than on algorithms, as exemplified below:

‘Using the algorithms is not sufficient in itself. You need to have an advanced level of training […] you have to be equipped enough with knowledge and then algorithms will come after.’ (HP4, A1-nurse, male, 58 yr.)

We did not observe physical algorithms being consulted, but several used laboratory tests to confirm a diagnostic suspicion from MH and PE. This fact was also expressed during interviews.

There were indications that HPs have a high reliance on laboratory tests for diagnosis. An A2-nurse expressed that he would always request laboratory tests after MH-taking and PE, and another one stated that he would use multiple laboratory tests to identify a disease, and if laboratory tests are negative, he could be lost. Similarly, an A1 nurse said that if a disease is not identified, she does further laboratory tests until identifying the problem. All COs also perceived laboratory tests as crucial for confirming a diagnostic suspicion.

### Perceived match between work function and educational background of healthcare providers

Several HPs found it valuable to work in the OPD, as it encompasses all services, and it is where you help most patients by making an initial diagnosis. In spite of this, HPs from all cadres believed that they carry a great responsibility in making diagnostic decisions. Clinical officers felt that the responsibility was rewarding as they manage patient- and community care, which they were taught to do. In contrast, there was a consensus that nurses adapt themselves to work beyond what they had trained for:

‘A nurse working in a hospital does what he/she should be doing while the same nurse working in a health centre does the work of a doctor, the work of a nurse, and the work of anybody […] Normally most of the things we do at the health centre, they fall into doctors’ responsibilities.’ (HP3, A2-nurse, male, 35 yr.)

Most A2- and A1-nurses felt that their tasks and responsibilities in the OPD at HCs matched those of physicians in hospital OPDs:

‘Consultation is not our job, it’s the doctor’s job but we do it because we are in health centres to help the population […] we have limited knowledge in this domain, but we do it.’ (HP1, A2-nurse, male, 33 yr.)

A2- and A1-nurses stated that their pre-graduate studies gave them the impression that they as nurses were to care for patients and assist physicians rather than independently carry out tasks and make decisions regarding diagnosis. They believed that this later role should have been emphasised:

‘If they said: “this [*diagnostic tasks*] will be included in your duties” you would also do this. We would have given much importance to it […] We were convinced that we were just receiving information for the sake of reading it, that our task was to take care of patients. Nothing else.’ (HP3, A2-nurse, male, 35 yr.)

### Educational training opportunities for healthcare providers

All A2-nurses believed that extensive training in conducting consultations was important for their capability to work in the OPD and found that the A2-level provided insufficient training in this. An A2-nurse claimed that the limited training in MH-taking and particularly PE triggered a lack of confidence in the diagnostic practice. A2-nurses also believed that the training was theory-driven with insufficient field experience in the diagnostic practice:

‘I don’t know if the one who designed the nursing curriculum had in mind that a nurse who leaves the school will immediately go, sit in the consultation and make decisions […] Considering the courses they learn, I think that they did not think about it a lot […] As we learn it theoretically, it’s difficult; things you only hear by means of your ears and coming from far, they are difficult to get.’ (HP3, A2-nurse, male, 35 yr.)

A1-nurses and COs agreed that A2-nurses are insufficiently trained for the diagnostic practice. However, one A2-nurse expressed that training in MH-taking was sufficient, and that internships gave him the confidence to conduct consultations:

‘[*Internships*] that’s the reason why what we learnt at that time was sufficient to the extent that you could leave the school and you immediately get into the service and work without any problems.’ (HP1, A2-nurse, male, 33 yr.)

The sense of responsibility seemed to increase with upgrading to A1-level, as there are cases at HCs that only A1-nurses manage. An A1-nurse expressed that the upgrading gave her the opportunity to increase her diagnostic competences, as she received pathology training during school and learnt how to diagnose. Furthermore, she had sufficient time for practise during internships. Yet, all A1-nurses found that the A1-level had deficiencies in preparing them for the diagnostic practice and managing cases, as pathology and learning how to conduct PE and interpret results from MH and PE were taught superficially:

‘They only provide training in issues related to public health, tuberculosis, and HIV/AIDS […] medical preparatory training is missing […] you are asking someone to do what you did not train them to do […] The curriculum does not allow a nurse to acquire skills related to conducting consultation.’ (HP4, A1-nurse, male, 58 yr.)

Clinical officers confirmed the nurses’ perspectives on insufficient training in the diagnostic practice:

‘Nurses don’t have the required knowledge, and this depends on the objective they set for you when you are studying.’ (HP9, CO, male, 37 yr.)

‘We realised that though we thought we knew things before [*at A1-level*], we did not know it […] you might sometimes recall some of the points you missed out in the diagnostics before because of the poor knowledge you had.’ (HP8, CO, female, 38 yr.)

Clinical officers emphasised that their educational training increased their confidence, as they received sufficient and high-quality training in clinical medicine and diagnostic practice. They also had much practical training in case management and much responsibility during internships, while collaborating with qualified supervisors.

A CO said that while nurses are trained for services other than consultation, CO training focussed primarily on diagnostic practice:

‘It [*CO education*] was of quality […] I think that there isn’t anything they didn’t show us concerning physical exams, or anything that we didn’t learn, especially because all those years we were only learning consultation […] What I appreciated more is history taking on which we were not emphasising in nursing. We couldn’t even understand its importance, and we did not put emphasis on physical exam too.’ (HP7, CO, female, 38 yr.)

### Perceived competences to perform diagnostic procedures

Although diagnostic responsibilities did not correspond well with nurses’ beliefs about what their work function should contain, A2- and A1-nurses expressed a high confidence in their ability to diagnose, while also expressing cautiousness:

‘Sometimes, we delay because we are hesitating because we don’t understand the case, and this might cause a problem.’ (HP5, A1-nurse, female, 51 yr.)

Another A1-nurse and all COs believed that they could manage patients as well as physicians. Clinical officers were generally very confident in the diagnostic practice:

‘Even if I might encounter a patient with a very difficult case, I manage to handle it.’ (HP8, CO, female, 38 yr.)

The high confidence was particularly evident for MH-taking. An A1-nurse believed that this came from knowledge and experience:

‘The confidence comes from the questions and from what you know. What you know orients good questions and then the patient will give you clear answers.’ (HP4, A1-nurse, male, 58 yr.)

Medical history-taking was only seen as difficult when patients gave partial or untrustworthy information. Most HPs were also confident in conducting a PE but found it difficult to interpret their findings:

‘I can do it [*PE*] and not find a diagnosis […] analysing or interpreting the results from physical exam is often difficult due to the lack of required skills and experience.’ (HP3, A2-nurse, male, 35 yr.)

Physical examination was especially difficult if cases were rare. Only one A1-nurse felt completely confident in doing PEs and using the information for determining preliminary diagnoses.

Some A2-nurses reported that they refer patients to the district hospital when they are unsure of the diagnosis (cf. Table 1). A1-nurses mentioned that it is highly important to be able to identify and refer patients who need referral:

‘Once we find that we are stuck, we transfer to the hospital. We don’t need to do things we are not able to while we have doctors at a higher level to help us.’ (HP5, A1-nurse, female, 51 yr.)

‘I can identify the patient who needs referring; I am able to detect it and to refer it if need be as soon as possible […] there are things I don’t master and this may push you to refer because you feel that you are unarmed, you feel that you have no orientation.’ (HP4, A1-nurse, male, 58 yr.)

### Resource constraints affecting ability to carry out work function

Clinical officers expressed that they only refer cases where resources and allowances at HCs are insufficient for proper diagnostic management.

The lack of consultation rooms and laboratory tests available at HCs was mentioned by several HPs from all cadres as resource constraints. They believed that this could compromise the ability to reach a diagnosis and force them to refer cases.

Another resource constraint mentioned by all HPs was time, because of a high number of patients (cf. Table 1) and a shortage of HPs. They expressed that they sometimes have to rush and prioritise procedures, which may lead to a poorer quality and inappropriate treatment:

‘[*W*]hen you receive a patient, they get in, they tell you what they have to tell you and then you immediately prescribe medicine or request exams without asking them anything […] This leads to a bad consultation […] when I am doing consultation, there are many things I don’t do while I should be doing them.’ (HP1, A2-nurse, male, 33 yr.)

Observations showed that the consultation time varied among nurses. All COs expressed that it is problematic that they have the training and competence to manage severe conditions and non-communicable disease, yet at HCs they are not using these skills as such cases are referred to the hospital:

‘We are ready to treat Rwandans, we are ready to do our job, we love our profession, but our structure needs to be recognised first.’ (HP9, CO, male, 37 yr.)

### In-service opportunities for healthcare providers to develop their professional competences

For both A2- and A1-nurses, the school training was not sufficient, but when they came to work in-service at HCs, they received training that made the diagnostic process easier:

‘We often had training [*in-service*]. Training helped us to increase our knowledge alongside the knowledge we got from school, and I know that we had sufficient training. This helped us to increase our capacity in making decisions.’ (HP3, A2-nurse, male, 35 yr.)

All HPs requested further in-service training opportunities and refresher-courses related to the diagnostic practice and management of the many health complaints within the OPD-service:

‘Normally things keep on changing; guidelines might change. However, there is nobody who is interested enough to say that we need refreshment. And say: “let us go and check if what those people are doing is up-to-date.” They just only stuck on what they gave us while we also need other supporting knowledge.’ (HP8, CO, female, 38 yr.)

Several HPs from all cadres declared that they improve their knowledge and skills in the diagnostic practice themselves, through searching the Internet, reading school books and reviewing cases they experienced. An A1-nurse expressed that the upgrading motivated him to increase his knowledge further, whereas an A2-nurse expressed that there was a lack of motivation to increase knowledge among A2-nurses:

‘There is a routine of negligence that we have. Normally, we do not update our knowledge; we don’t read […] I may say that we are simple lazy.’ (HP3, A2-nurse, male, 35 yr.)

All nurses and most COs had much experience from working at the HC and interacting with patients (cf. Table 1) and found that their diagnostic competences and self-confidence improved with this experience. All A2-nurses felt that work experience could compensate for their lack of knowledge from their educational training:

‘Right after I finished studying I did not know well different types of medicine, how I could do exams […] but as I went on working in the consultation for a long time, I got used to that and when I receive a patient in the consultation I am sure that I can treat any problem they have.’ (HP2, A2-nurse, male, 34 yr.)

One CO expressed that he did not get enough postgraduate experience in PE and MH-taking, which affected his capabilities:

‘When I consider the level at which we were after leaving school, had we continued practising, and had we had enough training, we would be at a good level, but I think we are stuck because we didn’t go to practise […] we didn’t immediately practise what we learnt.’ (HP7, CO, female, 38 yr.)

### Healthcare providers’ supportive work relations

All HPs addressed how peer collaboration and knowledge sharing helped them improve their diagnostic competences. For example, colleagues go for training regarding the diagnostic practice and pass on their knowledge to the rest of the staff by supporting them in the OPD-service. A2-nurses found it beneficial to ask more competent colleagues for help or observe them while working in the OPD-service:

‘We often do that [*consult with colleagues*] […] Because I can’t “do an adventure” and treat a disease I don’t know.’ (HP4, A1-nurse, male, 58 yr.)

From observations, nurses collaborated on a practical level. For instance, a nurse working in the child clinic helped the nurse working in the OPD-service after finishing with her patients.

One A2-nurse expressed that advice from colleagues may be questionable, as they may not have the proper competences in the diagnostic process and that knowledge sharing is not as beneficial as having had the training yourself.

Several HPs from all cadres described that external supervision from nurses working at hospitals was helpful. Supervisors come for check-ups and if they found something wrong, they provided recommendations for the staff to correct in their diagnostic practice. Healthcare providers expressed that this motivates them to improve their knowledge and performance. However, several HPs expressed that supervision is rare and short in time, and can be unfavourable and ineffective when focused on marks and checklists. An A1-nurse explained that this is because supervision is centred around performance-based financing (PBF):

‘The other kinds of supervision are political supervisions; those ones of PBF. They even give us marks; they just come and say: “I give you zero.” You just do it for the sake of making them happy so that they give you marks and then leave […] A good supervision is really the one which is formative. Yet, we don’t get those formative ones. In any way, they are not supervisions.’ (HP4, A1-nurse, male, 58 yr.)

Healthcare providers from all cadres affirmed that there is nothing to learn from such supervisions. Some nurses thought that it would be beneficial if there were frequent formative supervisions where HPs are trained by physicians:

‘If we had someone else like a doctor who would be here regularly and help us in those difficult cases we often face; we would treat those patients well because we don’t have sufficient knowledge.’ (HP2, A2-nurse, male, 34 yr.)

## Discussion

**FIGURE 1:**
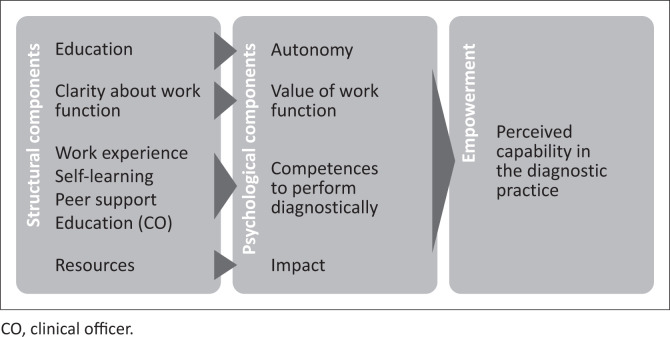
Structural components in the workplace influencing psychological components for empowerment in the diagnostic practice among healthcare providers at health centres in Rwanda.

While COs and nurses generally perceived themselves as competent in the diagnostic practice, findings indicate that other components influence their overall perceived capability to carry out procedures that are important for reaching a diagnosis, namely, dependency on diagnostic tools, educational background, match between work function and educational background, resource constraints at HCs, in-service learning opportunities and supportive work relations. This perception is comparable to the notion of empowerment in the workplace, which is defined as ‘a state where a person feels in control over own practice, enabling the person to competently fulfil professional responsibilities within an organisation’. Empowerment in the workplace is suggested to be a product of psychological and structural components.Psychological components are autonomy in decision-making, finding work function valuable, belief in own competences to fulfil work function and perform tasks skilfully, and ability to impact work.^28,29^ Psychological components are shaped by structural components in the workplace, such as clarity about work function, resource availability, opportunities for professional development, superior- and peer support, and the social composition of peers (in this case, educational level of HPs).^29^ Below, an attempt has been made to illustrate and explain the way that structural empowerment components influence psychological empowerment components and result in overall perceived empowerment in the diagnostic practice for study participants, cf. Figure 1.

### Autonomy in decision-making at different levels of educational and experience-based support

In this study, the educational level influenced HPs’ autonomy in the diagnostic practice. Clinical officers make autonomous decisions, whereas nurses’ decisions are supervised. Research shows that clinicians with much experience in PHC practice often recognise patterns and form a diagnostic hypothesis from presented patient complaints, without giving too much attention to other aspects of MH and PE.^5,30^ Healthcare providers who are less educated and less experienced will often gather much information about the patient from MH, PE and laboratory tests, before generating a hypothesis about a diagnosis.^30^ The high dependency on carrying out comprehensive MH-taking and PE for the HPs in our study may therefore imply an insecurity and lack of autonomy in the diagnostic decision-making, from a lack of both educational and experience-based support; thus, many participants claim to be experienced in the diagnostic practice.

In addition, several HPs expressed a view of laboratory tests as the mean through which to reach a diagnosis, rather than as a tool to rule out serious conditions informed by the MH and PE. Studies show that a high reliance on laboratory tests in PHC practice may pose problems of overusing tests and using laboratory tests as a screening tool. This consequently increases insecurity from inconclusive results from laboratory tests.^13,31^

Also, HPs used their own knowledge and judgement instead of relying solely on diagnostic algorithms, which indicate some degree of autonomy in diagnostic decision-making.

### Value of work function in task delegation

Clarity about work function and responsibilities can help to create a sense of meaning about one’s work function.^29^ While diagnostic procedures (MH, PE) were important to nurses, they felt that their responsibilities in the diagnostic practice were inappropriately beyond what they had been educated for, and what they believed their work function was about during their studies. In this sense, the discrepancy between education and responsibilities in the diagnostic practice, and the lack of clarity about responsibilities, may influence the value that nurses gave to their work function.

These findings comply with existing literature that suggest that many PHC-nurses in SSA carry out tasks beyond what their educational- and in-service training prepared them for.^2,4^ Literature suggests that task delegation to workers with a lower educational level can be empowering if responsibility and complexity in role and tasks are not too extensive.^29^ Furthermore, nurses can learn specific advanced skills that fall outside the scope of their routine practice and apply them in clinical settings.^1,2^ This was the case in Rwanda, where task delegation initiatives of HIV-services to nurses at HC-level have proven effective.^16,32,33^ Findings from our study suggest that task delegation within the diagnostic practice in OPDs at HCs in Rwanda is more complex. The OPD-service receives all types of patients and complaints, and problem-solving within the diagnostic practice must be adapted to deal with a multitude of problems. This requires a comprehensive set of skills and may make adequate, tailored training difficult.^30^ Yet, literature suggests that it is essential that primary HPs in LICs are appropriately trained for the tasks they are to undertake^1,2,33^ within the diagnostic practice.^14,34^

For COs, the opposite was the case. They felt competent to carry out responsibilities in the diagnostic practice from their education but were not able to utilise their competences fully in their work at HCs and did not believe that their profession was recognised. This may have compromised the value they gave to their work function.

### Competences from educational and in-service opportunities

HPs enhanced their competences in the diagnostic practice through in-service opportunities for professional development (work experience, self-learning and peer support), and nurses may be able to compensate for insufficient educational training through these opportunities. Promotion of formative supervision by competent supervisors, which include useful feedback and in-service training, was requested by HPs, as external supervision focussed on performance evaluations and marking for PBF. This fact and request corresponds with local research on supervision in PHC in Rwanda.^35,36^

While this was not the case for nurses, COs felt that their educational training enhanced their competences in the diagnostic practice, and A1-nurses also emphasised that educational upgrading was important. This aligns with studies showing that upgrading to at least a certified level (A1) is important for reaching a high level of empowerment and ensuring that HPs are competent in the diagnostic practice.^14,37^ The prioritisation of higher nursing degrees is common in many SSA countries,^4^ but upgrading may be challenged by having to work simultaneously with school. For this reason, an e-learning initiative was introduced in 2012 for the A1-nursing programme but inadequate Internet connection and information technology capacity hindered proper implementation, as students could not access the teaching forum.^19,38^

### Impact at work

Resource constraints such as time influenced HPs’ ability to perform diagnostic procedures optimally. Not being able to diagnose a patient may be perceived by HPs as not being able to impact one’s work. Nurses saw it as a cautiousness to refer patients that they were not able to diagnose to district hospitals, which according to both literature and the nurses is a strength and obligation to their practice.^30^ They may therefore not experience a lack of impact. Clinical officers, on the other hand, merely referred patients because of lack of resource availability and found it frustrating that they were not able to utilise their competences.

From this, there is an ambiguity in the perceived autonomy in decision-making, value of work function and impact at work among HPs, although all HPs seemed confident in own competences to perform diagnostically from in-service or educational opportunities. As all psychological components influence the overall perceived psychological empowerment,^28^ HPs may not feel fully empowered in the diagnostic practice, that is, capable of performing procedures important for competently reaching a diagnosis, with the resources available at Rwandan HCs.

### Strengths and limitations

The study was conducted in an inter-disciplinary, cross-country research team with local health professionals and collaborators to optimise contextualised data collection and interpretation. The interpretation of data was further strengthened by a comprehensive and iterative data analysis carried out by the researchers.

A limitation to the data collection methods was that the PI did not conduct the interviews because of language- and cultural barriers, which is usually the case in qualitative research. This made it necessary to have a local interviewer and a semi-detailed interview guide. It was not possible for the PI to probe on emerging questions during interviews, which created a risk of missing out on important insights from participants. To remedy the consequences of the PI not functioning as a data collection tool, a comprehensive discussion about preliminary findings with the interviewer was conducted, and the interview guide was adjusted accordingly after each interview.

Further, we did not conduct a long-term, regular, participatory observation with participants. Conclusions from observations were used keeping these limitations in mind.

This study is a qualitative study that addresses HPs’ perceived capability to carry out procedures important for reaching a diagnosis. Because of its qualitative nature, it does not evaluate the HPs’ actual performance in the diagnostic practice. Such quantitative data may be relevant to provide complementary evidence to this study, in determining and comparing performance of the three cadres of primary HPs in Rwanda.

### Implications and recommendations

The Rwandan health system may benefit from optimising the PHC workforce and practice, as this sector is essential to improve health service delivery and prevent morbidity and mortality. This exploratory study presents perspectives about HPs’ capability in the diagnostic practice at Rwandan HCs with the aim of generating useful knowledge for further research on how the PHC workforce and practice can be improved in Rwanda and other resource-constrained settings.

From our study findings, training COs to work in the OPD at HCs seems to be an appropriate response, as they perceive themselves as capable in the diagnostic practice. If school training is to improve capability within the diagnostic practice for A2- and A1-nurses, it needs to be tailored to fit these tasks. This study also suggests that in-service training, collaborative mentor schemes and formative supervision may be beneficial ways to improve perceived capability in the diagnostic practice for HPs. Therefore, future research may explore supportive in-service mentor schemes. This may be between HC-providers and physicians in hospitals.

There is a need for further research on how to improve the PHC workforce and practice in LIC, to advocate for political decisions to prioritise education or other empowering in-service initiatives.

## Conclusion

Nurses and COs working in the OPD at Rwandan HCs generally felt capable of carrying out procedures and make decisions in the diagnostic practice. Yet, nurses felt these tasks and responsibilities were beyond their professional training. COs believed that their professional training prepared them to function competently and autonomously in the diagnostic practice. HPs experienced in-service training and supervision as insufficient but increased their diagnostic competences by gaining experience at work, through self-learning and collaboration with peers. They were also highly dependent on the help from MH-taking, PE and laboratory tests in reaching a diagnosis but emphasised using own knowledge rather than algorithms. Resource constraints (time, rooms, laboratory tests) at HCs were seen as a barrier to perform diagnostic tasks optimally and to diagnose at the HC level.

Nurses may be able to compensate for insufficient school training from in-service opportunities, which implies that upgrading may be most effective through in-service training, collaborative mentor schemes and formative supervision. If school training is to improve capability within the diagnostic practice for nurses, it needs to be tailored to these tasks, as for the CO programme.
